# Women’s Preferences for Maternal Healthcare Services in Bangladesh: Evidence from a Discrete Choice Experiment

**DOI:** 10.3390/jcm8020132

**Published:** 2019-01-23

**Authors:** Rashidul Alam Mahumud, Nadia Ishrat Alamgir, Md. Tarek Hossain, Elaine Baruwa, Marufa Sultana, Jeff Gow, Khorshed Alam, Syed Masud Ahmed, Jahangir A.M. Khan

**Affiliations:** 1 Health Economics and Policy Research, Centre for Health, Informatics and Economic Research, University of Southern Queensland, Toowoomba, QLD 4350, Australia; gowj@usq.edu.au (J.G.); khorshed.alam@usq.edu.au (K.A.); 2 Health Economics and Financing Research, Health Systems and Population Studies Division, International Centre for Diarrhoeal Disease Research, Bangladesh (icddr,b), Dhaka 1212, Bangladesh; 3 BRAC James P Grant School of Public Health, BRAC University and Centre of Excellence for Universal Health Coverage, Dhaka 1212, Bangladesh; nishrat@bracu.ac.bd (N.I.A.); ahmed.sm@bracu.ac.bd (S.M.A.); 4 Maternal and Child Health Division, icddr,b, Dhaka 1212, Bangladesh; tarek.hossain@icddrb.org; 5 Abt Associates Inc., International Health Division, 6130 Executive Boulevard, Rockville, MD 20852, USA; Elaine_Baruwa@abtassoc.com; 6 Nutrition and Clinical Services Division, icddr,b, Dhaka 1212, Bangladesh; marufa@icddrb.org; 7 Deakin Health Economics, School of Health and Social Development, Deakin University, Burwood, Melbourne, VIC 3125, Australia; 8 School of Accounting, Economics and Finance, University of KwaZulu-Natal, Durban 4000, South Africa; 9 Liverpool School of Tropical Medicine, Pembroke Place, Liverpool L3 5QA, UK; Jahangir.Khan@lstmed.ac.uk

**Keywords:** discrete choice experiment, maternal healthcare, demand for healthcare, healthcare services, Bangladesh

## Abstract

Despite substantial improvements in several maternal health indicators, childbearing and birthing remain a dangerous experience for many women in Bangladesh. This study assessed the relative importance of maternal healthcare service characteristics to Bangladeshi women when choosing a health facility to deliver their babies. The study used a mixed-methods approach. Qualitative methods (expert interviews, focus group discussions) were initially employed to identify and develop the characteristics which most influence a women’s decision making when selecting a maternal health service facility. A discrete choice experiment (DCE) was then constructed to elicit women’s preferences. Women were shown choice scenarios representing hypothetical health facilities with nine attributes outlined. The women were then asked to rank the attributes they considered most important in the delivery of their future babies. A Hierarchical Bayes method was used to measure mean utility parameters. A total of 601 women completed the DCE survey. The model demonstrated significant predictive strength for actual facility choice for maternal health services. The most important attributes were the following: consistent access to a female doctor, the availability of branded drugs, respectful provider attitudes, a continuum of maternal healthcare including the availability of a C-section delivery and lesser waiting times. Attended maternal healthcare utilisation rates are low despite the access to primary healthcare facilities. Further implementation of quality improvements in maternal healthcare facilities should be prioritised.

## 1. Introduction

Maternal mortality in Bangladesh impacts inequities in access to primary health services with a huge gap between women in advantaged and disadvantaged socioeconomic communities [1]. About 800 women die per day due to preventable causes associated with reproductive health concerns [2]. Furthermore, the lifetime threat of maternal deaths related to childbearing was higher in remote areas and low resources settings [3]. According to the World Health Organization (WHO), about 99% of maternal deaths happen in low- and middle-income countries [4]. In 2015, the maternal mortality ratio (MMR) was 239 per 100,000 live births in low- and middle-income countries compared to 12 per 100,000 live births in high-income countries [4]. Bangladesh has in recent decades made significant achievements in maternal health indicators [5], with MMR being dramatically reduced [6,7,8]. Several initiatives undertaken by the government of Bangladesh includes introducing appropriate preventive strategies and effective collaboration with non-government organisations and other stakeholders. A number of initiatives such as emergency obstetrical care services and maternal health voucher schemes contributed significantly to improving primary healthcare outcomes [9]. However, this overall reduction does not accurately outline the whole picture of maternal healthcare status in the country [10]. Despite an increase in demand for maternal healthcare, limited supply and difficulties in access during pregnancy and delivery still leave a large number of women at higher risk for preventable death [11]. The individual demand for accessing quality maternal healthcare increases the maternal and child survival rates and significantly affects the mortality transition [5,12]. 

The provision of quality and adequate maternal healthcare services improve the experiences of pregnant women and outcomes for their babies. However, in low-resource settings, maternal healthcare services are critical to prevent adverse pregnancy outcomes (e.g., preterm birth, stillbirth, low birth weight) [13], which is significantly associated with high morbidity and mortality [14]. Demand-side barriers (e.g., geographical accessibility, availability, affordability, and acceptability) hamper women’s choice of healthcare services [15,16,17]. For example, information on healthcare services and/or providers [15], household resources and willingness to pay [15,16], opportunity costs, inequity of access (due to wealth, religious, political, ethnic characteristics), transportation system and community and cultural preferences, lack of health awareness, low self-esteem and little assertiveness [18,19] are the predominance of demand-side barriers. Similarly, service location, unqualified health workers, staff absenteeism, inadequate health services, costs and prices of services including informal payments, and staff interpersonal skills including trust may pose a significant supply-side barrier to accessing health services in low-resource countries including Bangladesh [15,16,17].

Interventions to improve women’s knowledge about accessible services and changing cultural attitudes can assist to increase the uptake of these life-saving services [16]. This increase in absorption is significant to achieve reductions in maternal and neonatal mortality rates [13]. This acceleration of uptake is critical to achieving the Sustainable Development Goals (SDGs) 2030 target 3.1 (reductions in MMR), 3.7 (ensure universal access to reproductive healthcare services) and 3.8 (achieve universal health coverage including access to quality essential healthcare services) [19]. Empirical evidence in the context of Bangladesh is limited in terms of women’s preferences for maternal healthcare services. As such, this study intended to address this gap by conducting a comprehensive discrete choice experiment (DCE). The DCE is a method elicit in health economics domain to examine patient’s preferences for various features of healthcare [20]. It consists of describing the service in terms of its attributes (e.g., outcomes or process measures) and associated attribute levels [21]. The significance of DCE over satisfaction surveys is that they take into account patients’ preferences for individual characteristics of healthcare [22]. This study also assessed the relative importance of maternal healthcare service characteristics to Bangladeshi women when choosing a health facility to deliver their baby. 

## 2. Materials and Methods 

### 2.1. Study Aim and Objectives

The objective of this study was to investigate the relative significance of the characteristics of maternal health services to Bangladeshi women by using the DCE method when choosing a health facility to deliver their baby. A hypothetical healthcare facility was thus developed to explore how different characteristics and levels of healthcare influence the demand for maternal healthcare services among Bangladeshi women. 

### 2.2. Study Setting

The study was conducted in four selected catchment areas of the NGO Health Service Delivery Project (NHSDP) in Smiling Sun (or Surjer Hashi) health facilities in Bangladesh. The Smiling Sun franchise program or network is funded by the United States Agency for International Development and the United Kingdom’s Department for International Development and is intended to complement government health facilities. The study research team worked with NHSDP staff to identify eligible households in the catchment area in four different settings: Harirampur (in the administrative district of Manikganj), Gazipur district, Keraniganj (in the Dhaka district) and Tejgoan areas (in Dhaka city)—all in the Dhaka Division of Bangladesh. 

### 2.3. Research Design 

**Figure 1:**
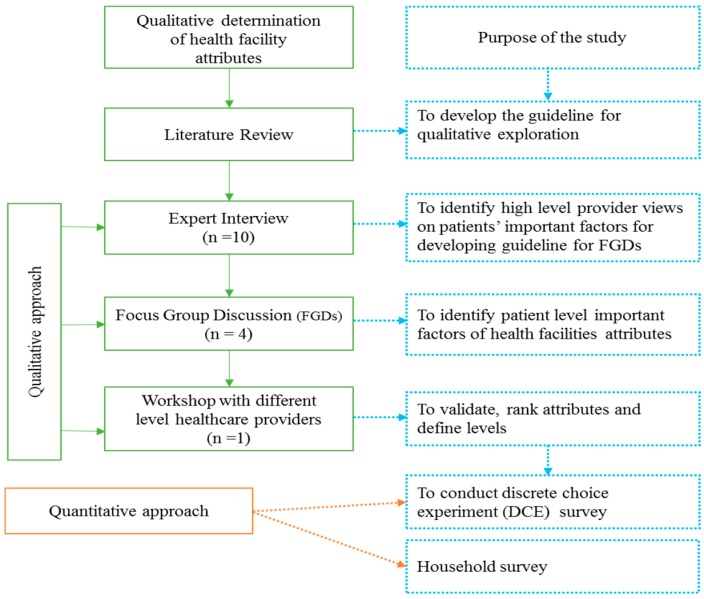
The study flowchart.

The study used a mixed-methods approach, i.e., both qualitative and quantitative. Qualitative methods (i.e., expert interviews, focus group discussions) were initially employed to identify and develop the characteristics, which influence women’s decision making most when selecting a maternal health service facility (Figure 1). These characteristics formed the basis for the quantitative methods (DCE and household survey) that were used to examine women’s preference for maternal healthcare services. Figure 1 below describes the methods that have been applied throughout the implementation of the study. 

### 2.4. Qualitative Approach 

#### 2.4.1. Literature Review

**Figure 2:**
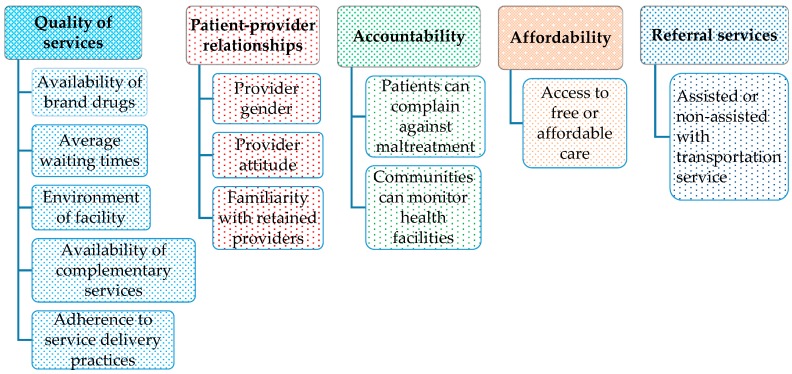
The attributes identified during the literature review.

The literature search strategy was designed to identify the most desired attributes of maternal health facilities including quality of services, patient-provider relationships, accountability, affordability and referral services (Figure 2). The attributes were categorized into five groups, which were further examined in expert interviews as well as in focus group discussions (FGDs). 

#### 2.4.2. Expert Interviews

An expert interview guideline was developed based on the findings from the literature review. A total of ten expert interviews were conducted among maternal and child health experts. The experts were selected from different organizations or institutes including: international research organizations (e.g., Save the Children, icddr,b), NGOs (e.g., BRAC, NGO Health Service Delivery Project), government institutes (e.g., Directorate General of Health Services and Directorate General of Family Planning, Ministry of Health and Family Welfare, Bangladesh), and medical research professional. Experts raised specific issues that they felt were likely to influence women’s preferences in choosing a health facility. Healthcare accountability, financing, and quality of services were common arguments with experts from government organisations or institutes, academia, and program implementers. However, healthcare financing was raised as a significant concern as financial hardship is an essential barrier to accessing healthcare. There have been recent initiatives such as vouchers, pay-for-performance schemes, and pre-payment mechanisms to reduce financial barriers to accessing healthcare. Accountability, in particular, community involvement, in the running health facilities was also perceived to be a significant issue. Other issues that were raised by experts included a designated referral system and the availability of healthcare and the associated distribution of sufficient human resources to meet demand.

#### 2.4.3. Focus Group Discussions

The guidelines for the Focus Group Discussions (FGDs) were developed based on the literature review and expert interviews in order to obtain information from potential survey respondents to validate the identified characteristics for the survey. The FGD guideline covered several topics: health-seeking behaviours, perceptions of the quality of healthcare and facilities, the main challenges of households’ appearance when in need of healthcare, and perceptions of health facilities and the payments for that. Four FGDs were conducted with 8 to 10 women in each to collate information for survey instrument development. Health service promoters organised these groups from four NHSDP clinic catchment areas. The participants of FGDs were excluded for the DCE and household surveys. Based on the participant’s responses, a structure content analysis was performed and re-structured by clustering within a similar group of maternal healthcare parameter. The main results arising were: (1) *Facility choice*: women select health facilities based on their health needs or demands, not based on distance or perceived health facility characteristics; (2) *Paying for services*: Respondents reported that they were satisfied in receiving healthcare services from the private facilities on payments or NGO clinics whereas they had to pay out-of-pocket and tended to prefer them over to public health facilities. The destitute mothers sought healthcare at public facilities generally because it is free but the quality of services was perceived to be higher at non-public facilities, which are chosen if payment could be made. Although the price of services is an issue, respondents traded it off in return for higher quality of services; (3) *Quality of care:* Participants also considered a broad range of health facility characteristics when they were asked how they thought of quality healthcare, such as immediate service, availability of healthcare providers (e.g., doctors/nurses), branded drugs and the availability of diagnostic services, and consistent attention and monitoring by staff during the time they are in the facility, courteous attitudes of facility staff, flexible opening hours, and the availability of referral or emergency services. 

#### 2.4.4. Final Workshop

Upon completion of expert interviews and FGDs, the research team had identified 15 attributes that appeared to be most influential for the utilisation of maternal health services. To review and prioritise the attributes, a final workshop was held in May 2014 with NHSDP staff including clinic managers and service promoters. The study method and preliminary findings were presented, and respondents were asked to review, rank and prioritise the attributes. In the DCE methodology, the characteristics of the health facility were referred to as attributes and the specific domains of those attributes or characteristics including attribute levels. The final set of nine attributes and their ranking are shown in Table 1. Given the high level of illiteracy among respondents, a pictorial guide was developed to represent the attribute and attribute levels. 

### 2.5. Quantitative Approach 

We conducted two cross-sectional surveys; a household survey to capture household-level characteristics and healthcare-seeking behaviour for maternal healthcare in 601 households and then the DCE survey among with its development. The DCE approach is a system of conjoint analyses or choice-based conjoint (CBC) analyses [21,23,24,25]. The CBC methods are effective in identifying preferences for services or non-market goods where the design contains information about the combinations of attribute levels to test for ensuring its efficiency [21,22,26,27,28]. The DCE method has been applied successfully to quantifying patient’s or client’s preferences in different health settings such as obstetric care [29], delivery care [30,31], cancer treatments [32,33], asthma medications [34], diabetes treatment and prevention [35], mental health [36], weight-loss programs [37]. The Sawtooth software was used to design the experiment so that the number of attribute levels was selected for the hypothetical choice set [38]. 

**Figure 3:**
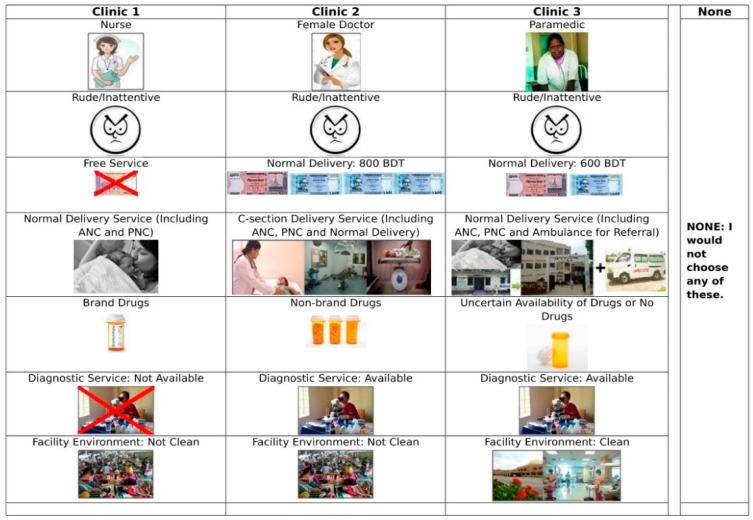
The hypothetical scenarios of the health facility.

A range of randomly generated hypothetical choice scenarios was developed whereas each level of the attribute has an equal chance of selecting in the choice set developed. A 12 different alternative versions of the DCE survey choice sets were designed, each having nine questions. An example of a single choice scenario presenting three hypothetical health facilities along with an option NONE, i.e., “I wouldn’t choose any of these”, is shown in Figure 3. Each option refers to a hypothetical health facility with seven attributes that were designated pictorially and with text in the local Bangla language and English version. Each hypothetical scenario shows six attributes, namely, a health facility with a service provider, attitude of providers, cost of service, continuum of care, availability of branded drugs, and availability of diagnostic services. The attributes for facility environment, availability for complaints, and waiting times were ‘cycled’ through so that only one was presented in each scenario, meaning that respondents could consider seven attributes at a time. The respondent was asked to observe the scenarios and select the most preferable one that denotes the facility they would choose. Before conducting the DCE survey, women reviewed the pictorial guideline (Table A1) and chose scenarios that explained three different hypothetical healthcare facilities using nine attributes. Eight of these attributes had answers that were selected in the dataset for analysis. One of the questions, nevertheless, was a ‘fixed’ select attribute signifying that the attributes in each of the three hypothetical scenarios shown in the scenarios were indistinguishable across the surveys that included a combination of attribute and attribute levels. The question was selected, one of the best options was a health facility fulfil all the attributes that could be considered most anticipated a priori. In this empirical experiment, the first facility scenario option considered a female medical doctor with a polite attitude, had free services, had branded drugs available, and a standard continuum of care from antenatal care to C-section delivery facility or referral with ambulance services. The fixed choice question was set so that each rational participant should choose this option, where this question was not an item that was unique or could be analysed. This system of the experiment process can be restructured if a high (<10%) percentage of participants perform irrationally or uncontrollably due to a lack of rationality.

### 2.6. Sample Size

A total of 566 individuals was required in the study. Assuming a 25% non-response rate, 588 participants were assessed, with 720 households visited, and 601 respondents agreed to participate in this study. Examining the equation for sample size provides an explanation, $N_{k}=\frac{\left(T_{k_{2}}\times \;SE_{k_{2}}\right)}{beta_{k}}$ where $N_{k}$ is the sample size, *T_k2_* is the t-statistic required for significance, *SE_k_* is the standard error for the prior parameter and *beta_k_* is the prior parameter [38]. Therefore, as beta approaches zero, the sample size needed to detect statistical significance increases. The sample participants were selected using the probability proportion sampling technique [39]. The probability of selection for a sampling unit was directly proportional to the size measure. The study participants were selected randomly in each catchment area from the eligible couples list of each facility. The design of the experiment was verified by using the Sawtooth Software to confirm an adequate sample size considering the number of attributes and attribute levels that were to be explored [40].

### 2.7. Data Collection Procedure

Data collection was completed during June–July 2014. The surveys were conducted with women aged 18 years or older who had delivered a baby in the past two years and had one or more child under five years. Written informed consent was taken from study participants and an explanation of the study aims and objectives was provided prior to the interview. Training was given to the data collectors on the objective of the study, confidentiality of information, respondent rights and techniques for conducting the interview. During data collection, checks were done by the study research supervisors to ensure the quality of the collected data.

### 2.8. Ethical Approval

This study protocol, discussion guides, and survey questionnaires were approved for human subject research from the Institutional Review Board (IRB) of BRAC James P Grant School of Public Health, BRAC University, Dhaka, Bangladesh and Abt Associates International (ethical reference no-34).

### 2.9. Estimation Strategy

#### 2.9.1. Descriptive Analysis

A descriptive univariate analysis for socio-demographic variables and a demand for maternal healthcare related variables were considered. Averages and percentages were showed to describe the study population including age, gender, socioeconomic status (SES), education status and health-seeking behaviours. Descriptive statistics were analysed using Stata 13 (StataCorp., College Station, TX, USA). The Sawtooth statistical package (Sawtooth Software Inc., Sequim, WA, USA) was used to measure individual utilities at the attribute level.

#### 2.9.2. Hierarchical Bayes Analysis

In the Choice-Based Conjoint (CBC) with Hierarchical Bayes (HB) estimation, it is hypothesized that individuals’ utility scores for all attribute are explained by using the multivariate statistical technique [40]. Choice-Based Conjoint with Hierarchical Bayes assumes that the participants’ responses choice sets based on a Multinomial Logit Model (MNL) [41]. MNL considers the probability of the specific alternative being chosen related to the proportion of the total utility for that concept relative to the total utility for all the concepts. This distribution is described using a mean vector and variance and co-variance matrix for an individual’s characteristics. At a minimum level, it is supposed that an individual’s probability of selecting particular alternatives are constituted by a multinomial logit regression model [41]. The Markov Chain Monte Carlo method was used to iteratively estimate the means and variance. Averages across all the participants are offered in the results section as ‘average utilities’ for each attribute.

## 3. Results

### 3.1. Background Characteristics 

Table 2 demonstrates the characteristics of the study population. A total of 720 eligible households were visited and 601 women agreed to participate in the study. Among these, 61% of the mothers were young adults aged (25 to 35 years); 27% had only completed primary education with 45% having completed secondary level and only a few (5%) had completed a higher secondary or higher level of education. Almost 50% of households consisted of 4 to 5 members, and 75% of mothers lived in urban communities. Around 57% of the mother’s households had a poor socio-economic status, while 24% belonged to the poorest group. 

### 3.2. Discrete Choice Experiment Results 

#### 3.2.1. DCE Attribute Rankings by Average Utility 

**Figure 4:**
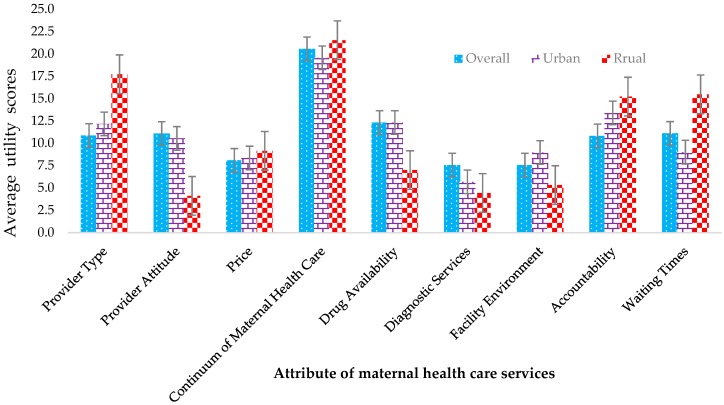
The distribution of discrete choice experiment (DCE) attribute rankings among the rural and urban participants.

Figure 4 shows the most desired attributes as expressed in utility scores for each attribute by the community location of the household. All attributes were ranked against all other attribute levels. Even though “utility scores” cannot be explained as an actual entity in and of itself, the utility scores are presented to give a direction of how much more required one attribute was, associative to another.

It was explained that a complete continuum of maternal healthcare including C-section was clearly the highest ranked attribute overall and among each of the rural and urban communities. Next, brand drug availability, provider type, polite providers, accountability and waiting time were clustered together overall in terms of utility, although some distinct variance exists across the wealth divide. Interestingly, a price required to obtain access to maternal facilities other than those provided by the government played a relatively insignificant role in the overall levels of satisfaction expressed.

#### 3.2.2. Distribution and Factors Influencing Higher Utility Scores for Maternal Health Services

Table 3 demonstrates the average utility scores for attributes by participant household location. An overall higher importance of attributes was: preference for female doctors, polite provider attitude, the price of normal delivery (up to 600 BDT), continuum of care including C-section, ANC, PNC, and brand drugs being available, and diagnostic service availability. The highest utility estimates were for drug availability, C-section service including ANC and a polite provider attitude. This indicates that those three attributes had a leading influence on women’s preferences for maternal healthcare services. These results are consistent with the HB results and show the statistical significance of almost all the preferred attributes outlined above.

## 4. Discussion

Bangladesh has made remarkable progress in decreasing maternal and child mortality and promoting women’s reproductive health over a couple of decades towards achieving Millennium Development Goals (MDGs) 4 and 5. However, women’s preferences for maternal healthcare services remains a growing concern. The utilisation of maternal healthcare services significantly influences the coverage of maternal healthcare. The primary objective of this study was to investigate the most significant attributes of maternal health services to Bangladeshi women when choosing a health facility to deliver their baby using the DCE method. The main findings of the study showed that women appreciated consistent access to a female doctor, the availability of branded drugs, respectful and attentive providers, a continuum of maternal healthcare including C-section service and low waiting time. Provider’s professionalism and dependable provision of quality care are the features that women appreciated most. 

Our study shows that the availability of a female medical doctor was one of the most significant attributes for women’s preferences to receive maternal healthcare compared to a male doctor. Some previous studies found that most women preferred receiving maternal healthcare services from female healthcare providers rather than a male provider [42,43,44]. This is due to several reasons including the fact that the majority of people are Muslim in the country; so, religious and cultural preferences are notable factors for receiving maternal healthcare from male doctors, which might be considered a religious sin as it is a gross violation of the veil [42]. Sometimes women may not prefer to share critical and complex medical information or conduct delivery with a male doctor [43,44]. Women perceived that if they receive maternity care from a male doctor, the “community people will not accept it simply rather they will insult women” [42]. People believe that their social reputation will diminish if delivery care is received from male doctors. A number of pregnant women feel ashamed to share their maternal health problems with male doctors and are afraid to receive delivery care at health facilities. A previous study conducted by Sychareun et al. (2012) in Lao People’s Democratic Republic, showed a consistent finding that some pregnant women feel shy and discomforted by having male healthcare facility providers, especially during delivery [45]. This research also confirmed that pregnant women disliked receiving maternal healthcare from male doctors due to the lack of privacy and confidentiality. As a result, the coverage of institutional delivery might be less appealing than home delivery. The availability of female doctors in health facilities ensuring comprehensive maternal healthcare should be considered an apparent technical significance [24,25,26,27,28,29,30,31,32,33,34,35,36,37,38,39,40,41,42,43,44,45,46,47,48], which might influence the optimum utilisation of maternal healthcare services in the country [47].

This study showed that women’s opinion about the competence of the respectful and attentive healthcare providers, availability of emergency maternity services, 24-h healthcare services, and promptness of care had significant influences on women’s decisions to utilise maternal healthcare services. The majority of the women expressed a desire for kind respectful medical providers at a health facility. However, unfriendly provider attitudes negatively affected women’s perception of the quality of maternity care [49]. Some studies found that respectful and attentive healthcare providers played a significant role in increasing the utilisation of maternal healthcare services at the health facility [47,48,49,50]. According to the WHO, healthcare programmes with a strong focus on respectful care are recommended to support changes in provider behaviours, clinical environments, and health systems to ensure that all women have access to respectful maternity healthcare services [51]. This study, in the same line of several previous studies, indicated that quality and respectful healthcare services were more important than distance and the cost of services to Bangladeshi women [50,52] and other developing countries [53,54]. The findings suggested that key stakeholders included healthcare providers, NGOs, and policymakers should frame a service protocol outlining appropriate and sensible attitudes for people seeking healthcare facilities. 

The study also found that the availability of branded drugs in the health facilities was significant of the women’s preference of selecting a facility. A Tanzanian study showed that implementing an intervention increased the awareness of the client’s knowledge about the availability of brand drugs [50]. That study suggested that steps should be taken about pricing and messaging of brand drugs to reduce the high proportion of women currently receiving curative maternal health services from informal providers. The findings from the FGDs and the DCE survey in this study displayed that free services were not desirable among women and that price or cost ranks lower than the quality of services they receive in importance. Nevertheless, it is significant to remember that if the urban or peri-urban setting study population differed drastically from the characteristics of rural women, it might not be justifiable to extrapolate the study findings to the rural settings. Similarly, it may be that the poorest of the poor in urban or peri-urban areas might not be the same as the poorest of the poor in rural areas. Health educational campaigns for healthcare professionals and clients might be an effective strategy for expanding the use of branded drugs for maternal healthcare services in developing countries like Bangladesh.

### Limitations

This study had some limitations. This study only considered women’s preference for the facility attributes and levels presented in the context of similar settings in Bangladesh and assumes no major facility attributes were omitted [53,55]. The study attributes were statistically significant as the expected facility preference showed a high equivalence with preference, assuming the limitations on the level of attributes and DCE design. The present study used a random design, which does not ensure that dominant choice sets do not occur or that there is a level balance. There are various features of study design that can influence the quality of data accumulated by a DCE and affect the analysis. The first of these encompasses the design of the choice experiment, including the choice of attributes and levels to be embraced and the structure of the choice scenario task. The second design issue involves the way in which attributes and attribute levels are described and presented in the choice tasks [19]. Moreover, the limited number of facility attributes analysed in any DCE by necessity excludes some structures that reflect a preference for some women. The findings of preferences for maternal healthcare facilities should be engaged as symptomatic rather than extrapolative (e.g., cultural, religious beliefs, social norms, and understanding of many Bangladeshi women are heterogeneous) of future maternal healthcare preferences. However, the study also experienced time constraints as women’s preference might be quite different now. This is because women’s preference of health facility attributes and levels were derived from the FGDs; women’s perceptions might vary over time. This study was conducted in the NHSDP catchment area in four different settings of Bangladesh that might influence the findings and may not be similar to other parts of Bangladesh. Awareness of diverse healthcare requirements in different locations and conditions is required [29,56]. Finally, the nature of the DCE study does not allow for exploring the causal inference of maternal healthcare demand. 

## 5. Conclusions

In this study, a DCE method was used to weigh women’s preferences for maternal healthcare facilities in Bangladesh. The findings support the notion that women maximise their utility from using health facilities by avoiding the poor-quality characteristics of maternal healthcare facilities. The reported women’s preferences are the only representative of the four specific areas surveyed, identified facility attributes and the significance of these values in provider characteristics might be shared by another group of women in low resource settings. Given the homogeneous nature of Bangladeshi society with esteem to economic status, culture and social norms, and language, the findings can be generalizable to the broader society with caution [56,57]. Women utilise maternal healthcare services in a facility, if it is staffed with encouraging, respectful healthcare providers and provide the required drugs. The packaging of facility attributes represents real-life decision-making that comprises a trade-off feature of the maternal health service between price and quality of available services. The findings identify a demand by women of reproductive age for improving the quality of maternal healthcare services at the primary level facility in Bangladesh.

## Figures and Tables

**Table 1:** The attributes of maternal healthcare services.

Attributes	Attribute Levels
**Availability of Provider**	1 = Paramedic
	2 = Nurse
	3 = Female Doctor
	4 = Male Doctor
**Provider Attitude**	1 = Polite
	2 = Rude
**Price**	1 = No fee
	2 = Normal delivery (≤600 BDT)
	3 = Normal delivery (≤800 BDT)
**Continuum of Maternal Healthcare (MHC) Services**	1 = No delivery service
	2 = Normal delivery + (ANC and PNC)
	3 = Normal delivery + (ANC, PNC and Referral)
	4 = Normal delivery + (ANC, PNC and Ambulance services for referral)
	5 = Normal delivery + (C-section, ANC, and PNC)
**Availability of Drug**	1 = Brand drugs
	2 = Non-brand drugs
	3 = Uncertain or no drugs
**Availability of Diagnostic Services**	1 = Yes
	2 = No
**Facility Environment Clean**	1 = Yes
	2 = No
**Availability for Complains**	1 = No option
	2 = Comment box
	3 = Assigned person
	4 = Phone line
**Waiting Times (in minutes)**	1 = <60
	2 = 60–120
	3 = >1204 = None

**Table 2:** The background characteristics of the discrete choice experiment (DCE) study participants (*N* = 601).

Variables	Mean (SD)/*n* (%)
**Average Age, Mean (SD)**	35.782 (10.969)
Age group in years	
19–24	25 (4.160)
25–35	369 (61.400)
36–45	122 (20.300)
46–64	65 (10.820)
≥65	20 (3.330)
**Education Background, *n* (%)**	
No education	143 (23.794)
Primary	160 (26.622)
Secondary	269 (44.759)
Higher secondary	21 (3.494)
Tertiary	8 (1.331)
**Household Size, *n* (%)**	
<4	184 (30.616)
4–5	298 (49.584)
≥6	119 (19.800)
**Household Main Occupation, *n* (%)**	
Farmer	22 (3.661)
Day Laborer	36 (5.990)
Service Holder	162 (26.955)
Business Owner	138 (22.962)
Rickshaw/Van/Boatman	51 (8.486)
Woodworker/ Foreman	48 (7.987)
Bus/Scooter Driver/Helper	59 (9.817)
Small/Micro Business Owner	50 (8.319)
Unemployed	5 (0.832)
Others	30 (4.992)
**Community, *n* (%)**	
Urban	453 (75.374)
Rural	148 (24.626)
**Socio-Economic Status, *n* (%)**	
Non-poor	116 (19.301)
Poor	341 (56.739)
Poorest	144 (23.960)

**Table 3:** The distribution of average scores ^1^ and factors influencing ^2^ maternal health services.

Attributes	All Participants		Community			
			Urban		Rural	
	Mean ± SD	Coefficient (SE)	Mean ± SD	Coefficient (SE)	Mean ± SD	Coefficient (SE)
**Availability of Provider**						
Paramedic	−6.693 ± 38.127	−0.067 (0.051)	−21.600 ± 35.867	−0.155 (0.06)	41.492 ± 48.796	0.277 (0.092)
Nurse	−22.370 ± 33.837	−0.149 (0.051)	−29.111 ± 41.732	−0.239 (0.059)	−26.407 ± 66.176	−0.195 (0.097)
Female doctor	29.639 ± 38.445	0.223 (0.048)	29.601 ± 36.527	0.252 (0.055)	−0.643 ± 64.586	−0.018 (0.096)
Male doctor	−0.576 ± 37.875	−0.007 (0.049)	21.112 ± 37.293	0.145 (0.056)	−14.441 ± 50.231	−0.064 (0.088)
**Provider Attitude**						
Polite	47.702 ± 32.891	0.335 (0.027)	45.267 ± 32.003	0.328 (0.031)	−2.042 ± 24.112	−0.004 (0.051)
Rude	−47.702 ± 32.891	−0.335 (0.027)	−45.267 ± 32.003	−0.328 (0.031)	2.042 ± 24.112	0.004 (0.051)
**Price**						
Free service	−2.073 ± 36.813	−0.055 (0.037)	−10.862 ± 32.133	−0.061 (0.043)	13.249 ± 33.301	0.081 (0.068)
Normal delivery (≤600 BDT)	1.259 ± 31.256	0.036 (0.036)	−9.932 ± 33.033	−0.06 (0.043)	−34.528 ± 28.508	−0.147 (0.069)
Normal delivery (≤800 BDT)	0.814 ± 37.527	0.018 (0.036)	20.794 ± 33.334	0.122 (0.041)	21.279 ± 29.856	0.066 (0.069)
**Availability of Continuum of MHC Services**						
No delivery service	−92.464 ± 65.582	−0.711 (0.065)	−85.145 ± 68.01	−0.734 (0.075)	30.196 ± 57.435	0.194 (0.105)
Normal delivery + (ANC and PNC)	−10.641 ± 42.318	−0.059 (0.055)	−0.263 ± 42.37	0.006 (0.063)	−15.542 ± 48.11	−0.087 (0.103)
Normal delivery + (ANC, PNC and Referral)	17.748 ± 41.368	0.052 (0.054)	27.069 ± 37.565	0.154 (0.063)	−17.832 ± 62.514	−0.276 (0.108)
Normal delivery + (ANC, PNC and Ambulance)	30.836 ± 33.955	0.268 (0.053)	21.336 ± 40.964	0.234 (0.061)	−19.643 ± 67.414	−0.064 (0.104)
Normal delivery + (C-section, ANC, PNC)	54.521 ± 39.482	0.449 (0.060)	37.004 ± 47.748	0.341 (0.068)	22.819 ± 98.579	0.234 (0.114)
**Availability of Drug**						
Brand drugs	55.076 ± 39.352	0.461 (0.036)	58.682 ± 35.787	0.512 (0.042)	20.177 ± 28.421	0.164 (0.071)
Non-brand drugs	−33.784 ± 30.481	−0.283 (0.038)	−33.142 ± 29.862	−0.316 (0.044)	−6.821 ± 24.371	0.006 (0.072)
Uncertain	−21.292 ± 32.847	−0.178 (0.037)	−25.539 ± 27.174	−0.196 (0.043)	−13.377 ± 27.001	−0.169 (0.074)
**Availability of Diagnostic Services**						
Yes	29.219 ± 24.754	0.253 (0.026)	19.513 ± 22.648	0.196 (0.03)	−5.357 ± 23.929	−0.005 (0.049)
No	−29.219 ± 24.754	−0.253 (0.026)	−19.513 ± 22.648	−0.196 (0.03)	5.357 ± 23.929	0.005 (0.049)
**Facility Environment Clean**						
*Yes*	25.772 ± 30.966	0.281 (0.046)	34.2 ± 33.837	0.288 (0.054)	−18.524 ± 23.582	−0.064 (0.083)
*No*	−25.772 ± 30.966	−0.281 (0.046)	−34.2 ± 33.837	−0.288 (0.054)	18.524 ± 23.582	0.064 (0.083)
**Availability for Complains**						
No option	−18.755 ± 41.247	−0.169 (0.081)	−31.904 ± 39.918	−0.337 (0.096)	28.528 ± 39.394	0.298 (0.157)
Comment box	7.086 ± 38.808	0.039 (0.083)	7.266 ± 53.211	0.023 (0.094)	11.681 ± 64.888	0.283 (0.162)
Assigned person	1.370 ± 34.466	0.008 (0.079)	21.396 ± 54.669	0.251 (0.088)	−23.651 ± 45.518	−0.413 (0.179)
Phone line	10.299 ± 40.617	0.121 (0.078)	3.243 ± 34.202	0.064 (0.091)	−16.558 ± 55.902	−0.168 (0.155)
**Waiting Times (in Minutes)**						
<60	5.185 ± 49.355	0.085 (0.063)	12.719 ± 40.61	0.184 (0.072)	−51.183 ± 45.092	−0.093 (0.116)
60–120	−6.805 ± 41.814	−0.071 (0.064)	3.064 ± 27.444	−0.036 (0.075)	−5.798 ± 41.189	−0.146 (0.115)
>120	1.620 ± 52.383	−0.015 (0.064)	−15.782 ± 40.773	−0.148 (0.075)	56.981 ± 55.803	0.239 (0.114)
None	8.898 ± 87.603	−0.321 (0.055)	5.264 ± 81.022	−0.368 (0.065)	−9.787 ± 62.002	−0.882 (0.129)

Note: ^1^ Average scores was derived using CBC/Hierarchical Bayes method, ^2^ Factors influencing higher utility scores using CBC/Logit model, BDT = Bangladesh Taka, SD = Standard Deviation, MHC = Maternal health service, ANC = Antenatal care, PNC = Postnatal care, C-section = Caesarean section.

**Table A1:**
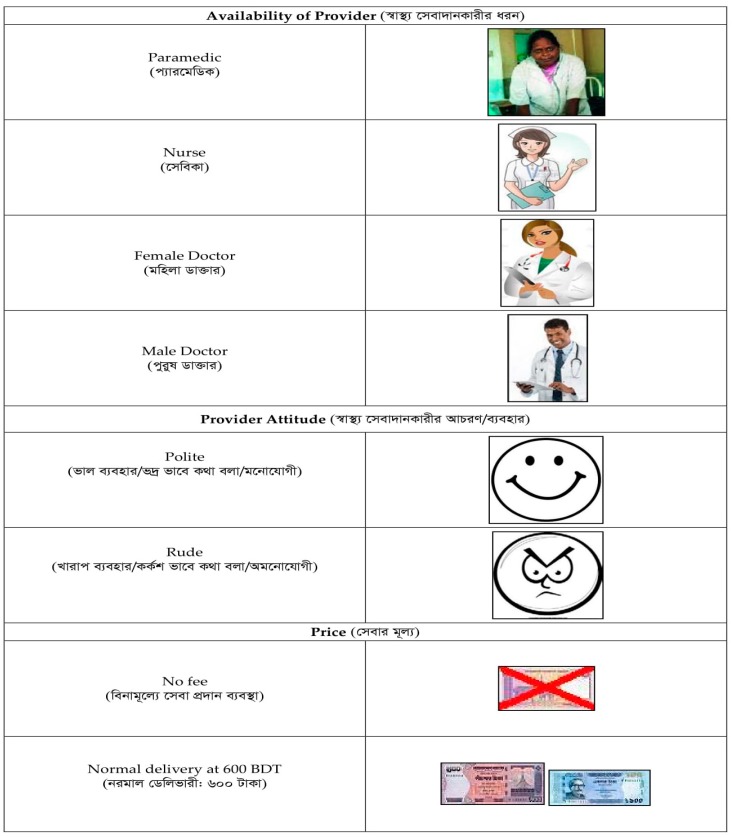

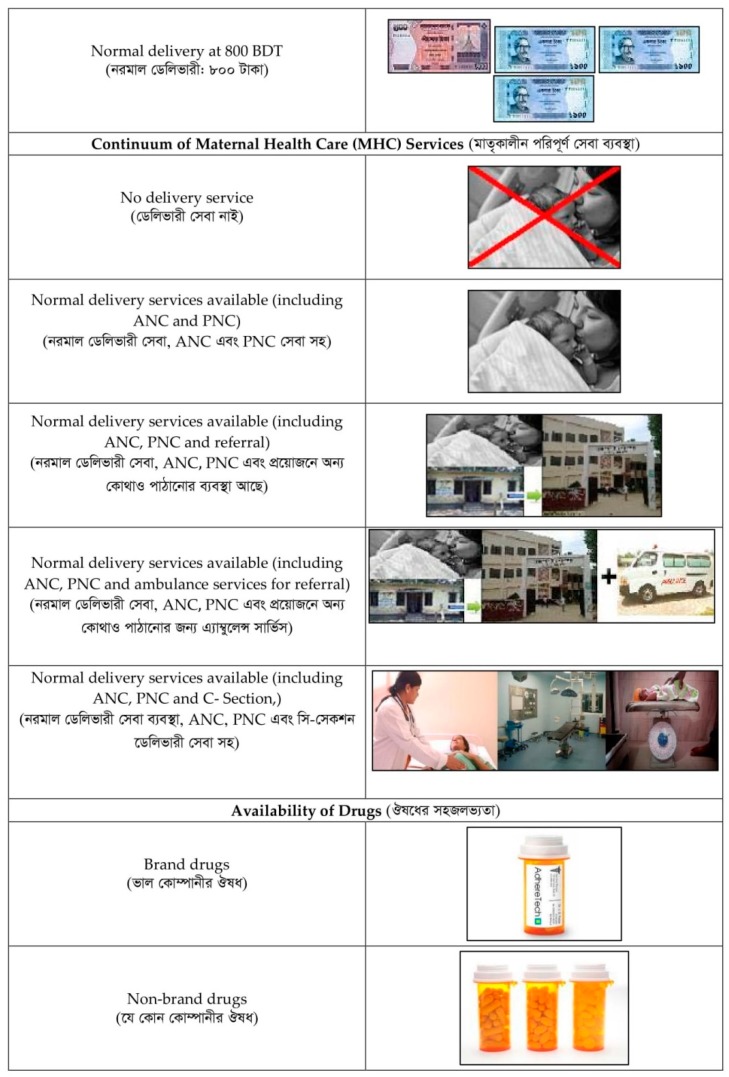

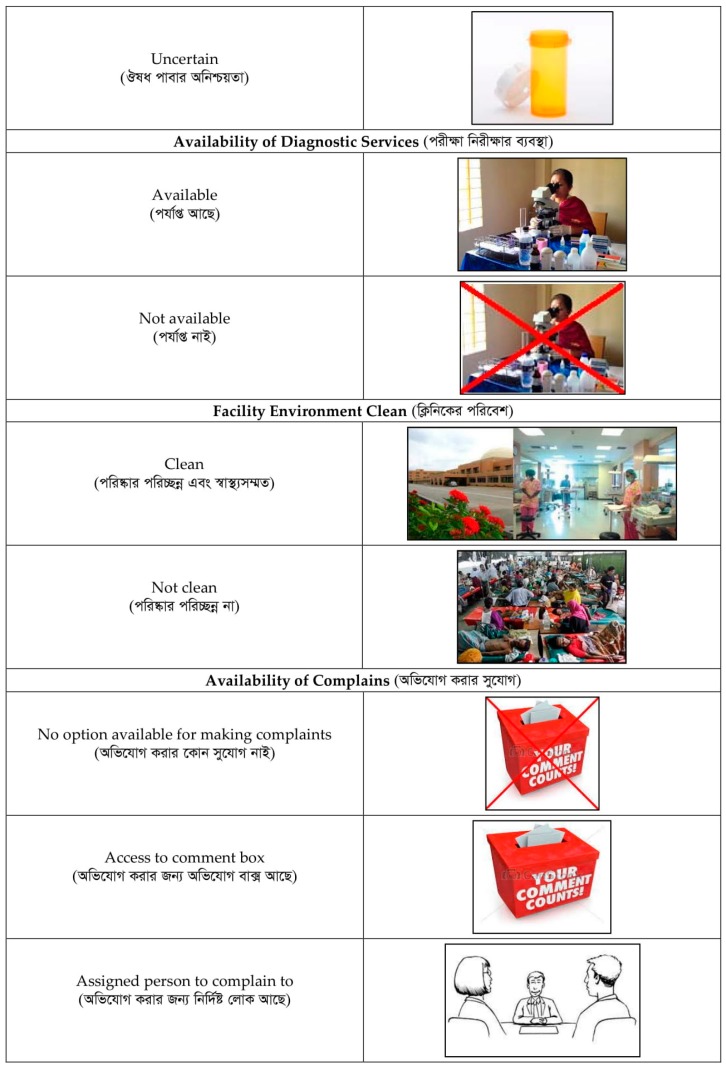

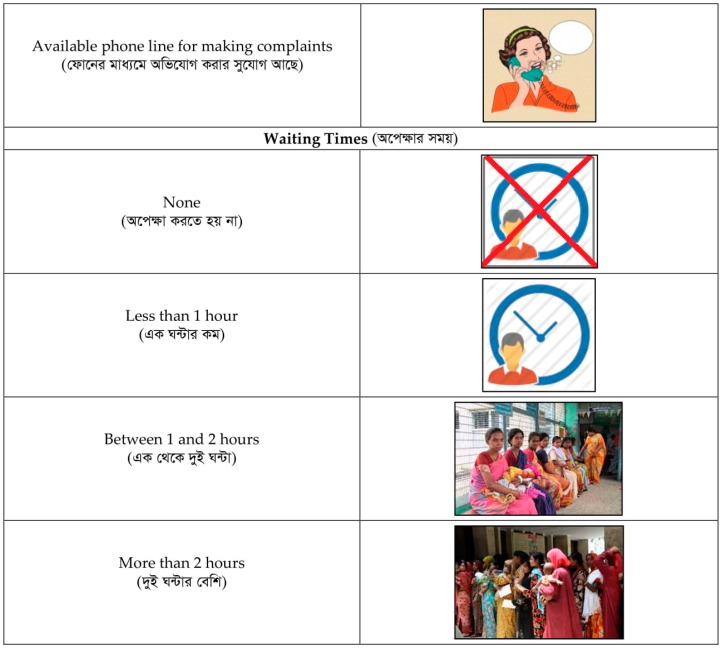
A pictorial guideline of the study attribute and attribute levels.

## Appendix A

Appendix was a brief explanation of the pictorial guideline of study attribute levels (Table A1). This guideline was used to develop the hypothetical choice scenario or clinic by the study research team. Before conducting the DCE survey, this pictorial guideline was explained to participants to enhance their understanding of the hypothetical scenario of choice set or clinic. It was also validated, reviewed and modified by ethical board members, researchers, maternal and child health experts and other medical professionals.
